# Genome-Wide Association Studies, Runs of Homozygosity Analysis, and Copy Number Variation Detection to Identify Reproduction-Related Genes in Bama Xiang Pigs

**DOI:** 10.3389/fvets.2022.892815

**Published:** 2022-05-31

**Authors:** Jiayuan Mo, Yujie Lu, Siran Zhu, Lingli Feng, Wenjing Qi, Xingfa Chen, Bingkun Xie, Baojian Chen, Ganqiu Lan, Jing Liang

**Affiliations:** ^1^College of Animal Science & Technology, Guangxi University, Nanning, China; ^2^Guangxi Key Laboratory of Livestock Genetic Improvement, Guangxi Institute of Animal Science, Nanning, China

**Keywords:** GWAS, ROH, CNV, litter size, teat number, candidate genes, Bama Xiang pig

## Abstract

Litter size and teat number are economically important traits in the porcine industry. However, the genetic mechanisms influencing these traits remain unknown. In this study, we analyzed the genetic basis of litter size and teat number in Bama Xiang pigs and evaluated the genomic inbreeding coefficients of this breed. We conducted a genome-wide association study to identify runs of homozygosity (ROH), and copy number variation (CNV) using the novel Illumina PorcineSNP50 BeadChip array in Bama Xiang pigs and annotated the related genes in significant single nucleotide polymorphisms and common copy number variation region (CCNVR). We calculated the ROH-based genomic inbreeding coefficients (*F*_ROH_) and the Spearman coefficient between *F*_ROH_ and reproduction traits. We completed a mixed linear model association analysis to identify the effect of high-frequency copy number variation (HCNVR; over 5%) on Bama Xiang pig reproductive traits using TASSEL software. Across eight chromosomes, we identified 29 significant single nucleotide polymorphisms, and 12 genes were considered important candidates for litter-size traits based on their vital roles in sperm structure, spermatogenesis, sperm function, ovarian or follicular function, and male/female infertility. We identified 9,322 ROHs; the litter-size traits had a significant negative correlation to *F*_ROH_. A total of 3,317 CNVs, 24 CCNVR, and 50 HCNVR were identified using cnvPartition and PennCNV. Eleven genes related to reproduction were identified in CCNVRs, including seven genes related to the testis and sperm function in CCNVR1 (chr1 from 311585283 to 315307620). Two candidate genes (*NEURL1* and *SH3PXD2A*) related to reproduction traits were identified in HCNVR34. The result suggests that these genes may improve the litter size of Bama Xiang by marker-assisted selection. However, attention should be paid to deter inbreeding in Bama Xiang pigs to conserve their genetic diversity.

## Introduction

Bama Xiang pigs, an indigenous Chinese pig breed, are famous for their excellent meat quality and early maturation (1). Owing to their small size, Bama Xiang pigs are easy to handle. Further, Bama Xiang pigs have anatomical and physiological traits similar to humans. Therefore, these pigs could be used in human medical research, such as hypertrophic scarring and diabetes (2, 3). However, genetic diversity in Bama Xiang pigs is declining due to historical inbreeding. Runs of homozygosity (ROH) arise when the same haplotypes are inherited from parents (4), especially in inbreeding Bama Xiang pigs. Bama Xiang boars sexually mature in 76 days and often mate with their mothers resulting in inbreeding (5). The increased inbreeding and declining genetic diversity may hamper the sustainable production of the Bama Xiang pig. Moreover, replacing traditional breeding with intensive pig farming has been beneficial to Bama Xiang pig breeding. The ROH analysis and evaluation of inbreeding rates are important for conserving Bama Xiang pig resources.

Copy number variations (CNVs) are a subtype of genomic structural variation ranging from 50 bp to several Mb in length. The copy number variation region (CNVR) is the area adjacent to the copy number with overlapping regions (6). The CNVR, owing to its length, has a higher probability of changing gene structure and gene dosage and is known to affect several traits in pigs. Qiu et al. demonstrated that nine CNVRs were associated with average daily gain and days to 100 kg in Duroc (7). Zheng et al. (8) reported that the copy number of the *AHR* had a positive effect on reproduction traits. The copy number variation in *GPER1* might be related to the litter size in the Large White pig breed (9). Bovo et al. (10) showed that the CNVR in *MSRB3* may be associated with the ear size. Thus, analyzing CNVR function has become an important part of porcine genetics and breeding.

Single nucleotide polymorphism (SNP) arrays can genotype hundreds of thousands of SNPs distributed throughout the genome (11). Based on the density, the porcine SNP arrays were split into 50K (12), 60K (13), and 80K (14). The beadchips have been widely used for genomic selection (15), selection signature research (14), and genome-wide association studies (16). The array can be used to complete population genetics research (17) and CNV detection (18).

Litter size and teat number, which are the base index, are typically associated with economic benefits and production ability in the porcine industry. While the teat number has a medium level of heritability, the litter-size heritability is low. During breeding, the teat number trait increases with an increase in the litter-size trait (19, 20). Litter size represents productivity levels per sow per year in pigs (21). Teat number is a proxy for lactation ability and, thus, is related to piglet mortality rates (22). Numerous researchers have focused on identifying SNPs, quantitative trait loci (QTLs), and candidate genes associated with litter size and teat number (23–25). However, the studies of quantitative traits based on CNVs have rarely been completed, especially on litter size and teat number. Therefore, a study on CNV associated with litter size and teat number in pigs is required.

In this study, we completed the genome-wide association study (GWAS) and the ROH analysis to evaluate the genomic inbreeding coefficients (*F*_ROH_) of Bama Xiang pigs using SNP array data. We also performed CNV detection and CNVR-based association analysis of litter size and teat number of Bama Xiang pigs. We identified the genes in significant SNPs, ROH, and CNVR and provided the candidate genes associated with litter size and the teat number of Bama Xiang pigs. The information on the Bama Xiang pig can provide the development of the molecular mechanisms of litter size and teat number. Understanding the genetic basis of litter size and teat number in Bama Xiang pigs should help us improve their reproductive capacity.

## Materials and Methods

### Animal and Phenotype Data

This study collected ear tissues of 403 Bama Xiang sows from the Agriculture and Animal Husbandry Co. Ltd. (Guangxi, China) for genomic DNA extraction. Parity and teat number data from 297 sows (2,199 dens) were also obtained to calculate phenotype statistics of 14 litter-size traits and five teat-number traits (Supplementary Table S1).

### The DNA Subjects and Genotyping

Using a tissue DNA isolation mini kit (Vazyme, Nanjing, Jiangsu, China, Cat. #DC112-01), the genomic DNA was isolated from the ear tissue. Porcine SNP50 BeadChip (Illumina, Inc.) containing 51,315 SNPs was used to genotype the genomic DNA. The raw data were called using the GenomeStudio 2.0 software. Single nucleotide polymorphism arrays with call rates less than 0.9 or with minor allele frequencies of <0.05 were removed in PLINK, version 1.90. Single nucleotide polymorphism arrays without location information were also deleted. After quality control, 403 sows and 24,123 autosomal SNPs were used for analysis. Beagle, version 5.0, was used to impute missing alleles.

### Genome-Wide Association Studies

For each trait, we implemented GWAS using a univariate linear mixed model in GEMMA. The GWAS model was as follows (26):


$$\begin{array}{l} \text{y}\;=\;\text{W}\alpha \;+\;\text{x}\beta \;+\;\text{u}\;+\;\varepsilon ;\;\text{u}\;\sim \text{ MVNn}\left(0,\;\lambda \tau^{-1}\text{K}\right),\;\varepsilon \;\\ \sim \text{ MVNn }\left(0,\;\tau^{-1}\text{I}\text{n}\right), \end{array}$$

where **y** is an *n*-vector of reproduction traits, **W** is a matrix of fixed effects including three principal constituents and the parity effect, **x** is the SNP genotype, α and β are the corresponding coefficients, **u** is the random effect, **ε** is the random error, τ^−1^ is the variance of residual errors, λ is the ratio between the two variance components, **K** is the kinship matrix, **In** is the *n* × *n* identity matrix, and MVN*n* denotes the *n*-dimensional multivariate normal distribution. Thresholds for Bonferroni-adjusted genome-wide significance and suggestive significance were defined as –log_10_(*p*) = 5.68 (0.05/24,123) and –log_10_(*p*) = 4.38 (1/24,123), respectively (27–29). We defined the 200 kb regions upstream and downstream of the saliency marker as significant for identifying genes in the Biomart program (*Sus scrofa 10.2*).

### The ROH Detection and ROH-Based Genomic Inbreeding Coefficients

The ROH was identified using detectRUNS packages with the following parameters: Window size was 15; the threshold was 0.05; the minimum number of homozygous/heterozygous SNP in the window was 30; the maximum number of SNP with opposite genotype was 1; the maximum number of missing genotypes was 1; the maximum gap between consecutive SNPs was 250,000; minimum length of the run was 1,000,000; and number of SNPs every kilo-basepairs was 1/100. The ROH length was classified into four classes: 0–6, 6–12, 12–24, and 24–48 Mb. The *F*_ROH_ for Bama Xiang pigs was estimated using the following formula (30):


(1) $$\begin{array}{r} F_{\text{ROH}}\;=\;L_{\text{ROH}}/L_{\text{AUTO}}, \end{array}$$

where *L*_ROH_ is the total length of ROH on autosomes and *L*_AUTO_ is the total length of the autosomes.

The relationship between *F*_ROH_ and Bama Xiang pig reproduction traits was calculated using the Spearman coefficient. The SPSS 19.0 was used to calculate the general lines model.

### The CNV Detection and CNVR Annotation

The CNVs were detected using cnvPartition 3.2.0 and PennCNV 1.0.5 software. As a plug-in software in GenomeStudio 2.0, cnvPartition detected different copy numbers using Gaussian distribution by Log*R* ratio and B allele frequency values. To increase the accuracy of CNVs, the cnvPartition was completed with the following parameters: Confidence threshold, 35; minimum homozygous region size, 1,000,000; and minimum probe count, 3. PennCNV used the Log*R* ratio and B allele frequency values to identify the copy number base on the hidden Markov model with parameters: numSNP, 3; length, 1 K. The CNVs were detected using cnvPartition, and PennCNV was merged into CNVRs using bedtools software. According to the types of CNVRs, we merged the same type of CNVR from two software into CCNVR. The CCNVR of Bama Xiang pigs was annotated using the BioMart program and David database. The Pig QTL database (https://www.animalgenome.org/cgi-bin/QTLdb/SS/index) was used to annotate the traits related to the CCNVR.

### The CNVR-Based Association Analysis

According to the CNVR frequency detected by cnvPartition and PennCNV, we defined the CNVR frequency over 5% as the HCNVR. To identify the effect of HCNVR on Bama Xiang pigs' reproductive traits, we completed a mixed linear model association analysis with the Q + K method using TASSEL software, where Q is the principal component matrix and K is the kinship matrix. The Q and K matrices were calculated by SNP array data using Tassel software. According to the Bonferroni thresholds of GWAS, we defined *p* = 0.001 (0.05/50) and *p* = 0.02 (1/50) as the significance thresholds and suggestive significance thresholds, respectively (27–29). The significance and suggestive significance HCNVRs were annotated using the BioMart program.

## Result

### The Significant SNPs and Genes From GWAS

**Figure 1:**
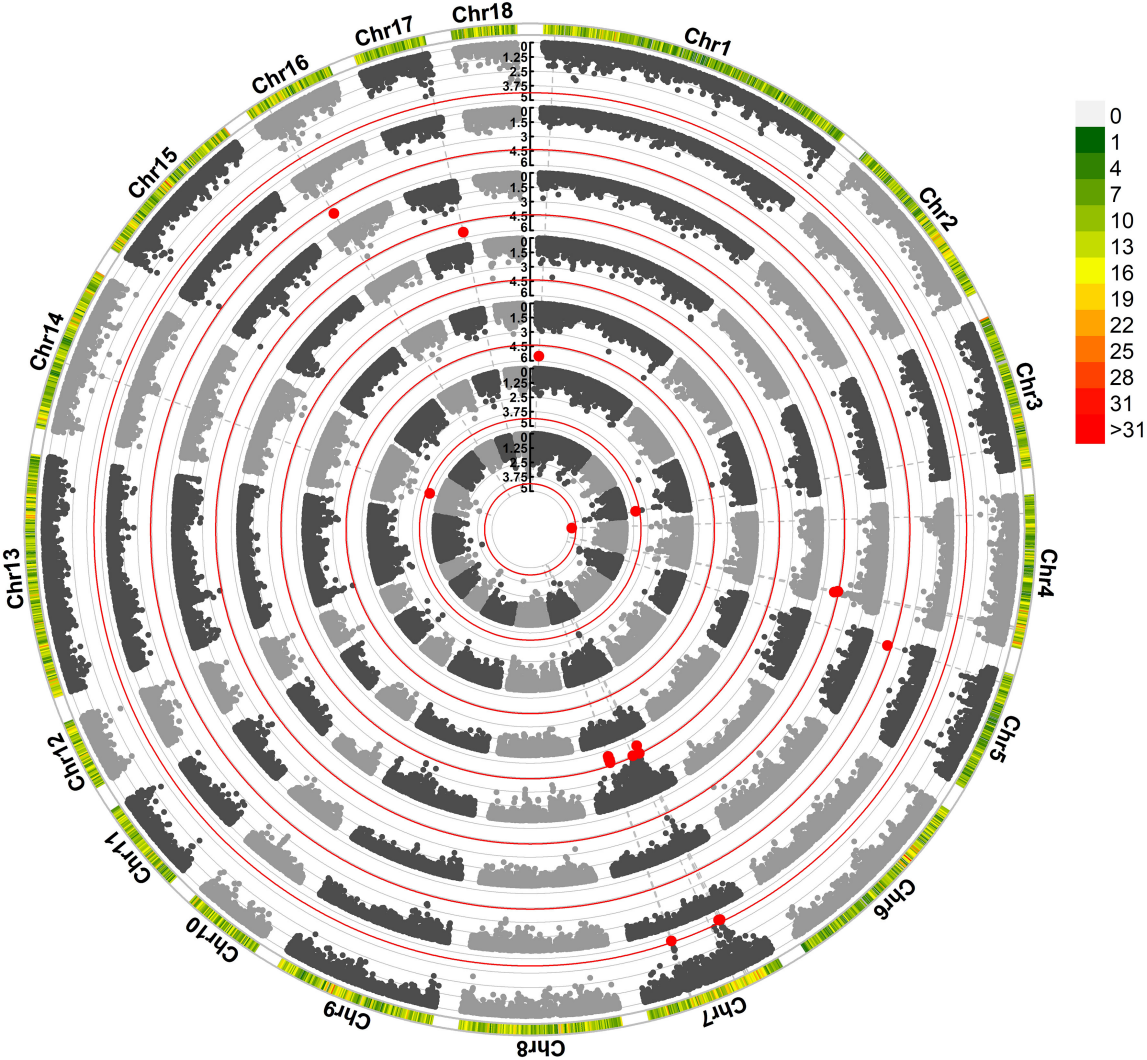
Manhattan plots obtained in the GWAS of litter-size traits. The red line identifies the cut-off for suggestive significance. Red spots identify SNPs with suggestive significance. Traits, from the inner to outer lanes are average birth number (ABN), birth number–second (BN2), birth number–fourth (BN4), birth number–fifth (BN5), birth number–ninth (BN9), and maximum number of births.

We found that 29 SNPs exceeded the suggestive significance threshold in SSC 1, 3, 4, 5, 7, 14, 16, and 17. Seven litter-size traits (Figure 1) and four teat-number traits (Supplementary Figure S1; Supplementary Table S2) were covered, including average birth number (ABN, one SNP), birth number–second (BN2, two SNPs), birth number–fourth (BN4, one SNPs), birth number–fifth (BN5, 10 SNPs), birth number–sixth (BN6, four SNPs), birth number–ninth (BN9, two SNPs), the maximum number of births (MAXBN, three SNPs), teat numbers on the left side (LTN, one SNP), teat numbers on the right side (RTN, one SNP), the total number of teats (TTN, three SNPs), and minimum teat number (MINTN, one SNP). The SNP rs80960023 on SSC4 was significant in ABN. The SNP rs80967544 was a significant SNP in BN5 and MAXBN. The SNP rs81450533 on SSC14 was significant in LTN, TTN, and MINTN. Forty-seven genes were significantly associated with litter-size traits (Supplementary Table S3). Notably, 12 genes were identified as related to reproduction, including *CIB4, DRC1, HADHA, DMC1, DDX17, RAB23, HMGA1, SLC26A8, MAPK14, ARRDC4, CDH6*, and *BMP7*.

### The ROH Detection and Genomic Inbreeding Coefficients Evaluation

We detected 9,322 ROHs, with an average length of 88.78 Mb, in 403 Bama Xiang pigs. The number of ROH per animal ranged from 1 to 136, with the average number of ROH being 23.14 ± 13.74 (mean ± SD). The number of ROH for 0–6, 6–12, 12–24, and 24–48 Mb was 8,369 (89.77%), 831 (8.91%), 110 (1.18%), and 12 (0.13%), respectively. The genomic inbreeding coefficients per sow ranged from 0.0013 to 0.24, with the average genomic inbreeding coefficient as 0.036 ± 0.024 (mean ± SD). A total of 12 litter-size traits had a significantly negative relation with *F*_ROH_ (*p* < 0.05), including ABN (−0.28), BN1 (−0.19), BN2 (−0.19), BN3 (−0.25), BN4 (−0.15), BN5 (−0.20), BN6 (−0.23), BN8 (−0.17), BN9 (−0.22), MAXBN (−0.22), and the minimum numbers of birth (MINBN) (−0.25) (Supplementary Table S4). The general linear model about ABN and *F*_ROH_ was ABN = 10.359–15.875 ^*^F_ROH_, with *R*^2^ was 0.62, and *p* < 0.05.

### The CNV Detection and Annotation

**Figure 2:**
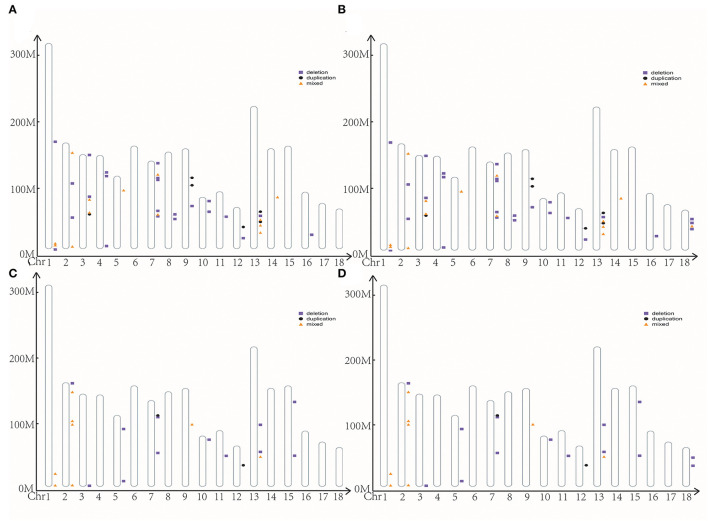
The position of CNVR. **(A)** CNVR from cnvPartition; **(B)** CNVR from PennCNV; **(C)** the common CNVR; **(D)** the high frequency CNVR.

We identified 2,920 and 397 CNVs using cnvPartition and PennCNV software, respectively. After the merge, we removed the length of CNVRs over 4 Mb. Finally, we got 197 CNVRs ranging from 0.036 to 3.72 Mb, including 159 deletions, 10 duplications, and 28 mixed in cnvPartition (Figure 2A). We got 45 CNVRs ranging from 0.093 to 2.97 Mb, including 26 deletions, six duplications, and 13 mixed in PennCNV (Figure 2B). We merged the CNVRs from cnvPartition and PennCNV based on their type. Finally, we got 24 CCNVR, including 11 deletions, one duplication, and 12 mixed (Figure 2C; Supplementary Table S5). A total of 64 genes were located in CCNVRs. The genes were significantly enriched (*p* < 0.05) in the cilium movement and outer dynein arm assembly pathway. We located 637 QTLs in CCNVRs, including 26 average backfat thickness QTLs, 21 backfat at last rib QTLs, and 17 loin muscle area QTLs (Supplementary Figure S2).

### The CNVRs Associated With Reproduction Traits

With cnvPartition and PennCNV software, we got 242 CNVRs in Bama Xiang pigs, including 50 HCNVR (Figure 2D; Supplementary Table S6). After execution of the association analysis, four HCNVRs were over the significance thresholds, and nine HCNVRs were over the suggestive significance thresholds. Interestingly, HCNVR10 (chr2 from 152455383 to 152748172) was over the suggestive significance thresholds in ABN and BN4, BN5, and MINBN (Supplementary Table S7). Additionally, HCNVR34 (chr14 from 124353052 to 124685417) was over the suggestive significance thresholds in ABN and MAXBN, the maximum number of teats (MAXTN), TTN, and LTN (Supplementary Table S8). We executed a *t*-test with reproduction traits between different CNVR types in HCNVR10 and HCNVR34 (Supplementary Table S8). The deletions were lower than normal in ABN, BN4, BN7, MINBN, and other reproductive traits such as HCNVR10. In ABN, MAXBN, MAXTN, TTN, and LTN, the phenotype of deletions was lower than normal in HCNVR34. After annotation, we got two genes (*NEURL1* and *SH3PXD2A*) related to reproduction traits located in HCNVR34.

## Discussion

### The GWAS Reveals the Candidate Genes Related to Litter Size and Teat Number

We identified 18 genes by using GWAS. Of these, *MAPK14* was the only gene that was not associated with a specific sex; *MAPK14* promotes phosphorylation and is therefore involved in both male and female reproduction (31). It is critical for the heat-induced proliferation of spermatogenic cells. Moreover, *MAPK14* is mainly localized in granulosa and follicle cells in the ovaries, implying its importance during follicular development (32).

Most of the remaining genes were related to sperm structure, spermatogenesis, sperm function, and male infertility. Gene *CIB4*, which is strongly expressed in mouse and human testes, plays an essential role in the spermatid head. Gene *CIB4* knockout (KO) mice are sterile because haploid differentiation becomes impaired (33). Knocking out the *DRC1* gene in mice completely disorders the axoneme structure of sperm flagella, impairing sperm motility (34). Research in bulls shows that *HADHA* proteins are significantly more abundant in the immotile sperm of low-fertility males than in the sperm from high-fertility males (35). Gene *DMC1* plays a vital role in repairing DNA double-strand breaks, and disruption of this repair process is linked to male infertility (36). Gene *DDX17* increases during the transition from spermatocytes to sperm (37). Research in a Duroc × Erhualian F2 population identified *RAB23* as a candidate gene for pubertal reproductive failure (38). In humans, *HMGA1* is a stage-specific marker gene for germ cells, and in mice, it is essential for sperm production (39). Gene *SLC26A8* is a sperm-specific member of the *SLC26* family, and its heterozygous missense mutations are highly associated (power of >95%) with asthenozoospermia (40). Gene *ARRDC*4 mediates extracellular vesicle biogenesis, which appears to be required for sperm function, given that *ARRDC4* KO mice have impaired sperm (41). Two of the 18 genes were related to ovaries, follicles, and female infertility. Gen *CDH6* regulates endometrial adhesion and implantation. Studies show that the *CDH6* gene is dysregulated in the endometrium of women with infertility (42). Gene *BMP7* regulates steroidogenesis, granulosa cell states, and follicular development. A study on Yorkshire pigs found that it is a candidate gene for litter size (43, 44).

### The ROHs Reveal the *F*_ROH_ Effect on the Litter Size

In this study, we successfully identified 9,322 ROHs. Similar to Laiwu pig (45), Diannan xiaoer pig (46) and Large White (47), the majority ROHs in Bama Xiang pigs were short segments. The significant Spearman correlation coefficient of *F*_ROH_ and litter size ranged from −0.14 to −0.28. No significant correlation was noticed between the *F*_ROH_ and teat number. Our result, same as proposed by Tao et al. (48), illustrated that the inbreeding had a significant effect on the litter size but had no effect on the teat number in Bama Xiang pigs. According to the general linear model, the ABN decreased to 0.16 when the *F*_ROH_ was increased to 0.01. Thus, the average number of births decreased to 0.57 ± 0.38 in Bama Xiang pig. We established that decreased *F*_ROH_ improves the litter size in Bama Xiang pigs.

### The CNV Reveals the Candidate Genes Related to Litter Size and Teat Number

We identified 3,317 CNVs, 242 CNVRs, and 13 CCNVRs in Bama Xiang pigs. A total of 11 genes related to reproduction, *TUBB4B* (49), *STPG3* (50), *SNRPA1* (51), *NRARP* (52), *NEUROD4* (53), *MGAT1* (54), *LRGUK* (55), *LCN8* (56), *LCN12* (57), *LCN10* (58), and *CCDC183* (58), were identified in CCNVR. Bama Xiang pigs are famous for their excellent meat quality and early maturation; however, their lower growth rate and higher backfat content make them less desirable. Thus, the result that the top 10 QTLs in CCNVRs are major related to backfat, meat quality, growth, and teat number is reasonable. However, reproduction-related QTLs were not found in CCNVR1. After executing association analysis, we discovered that HCNVR10 and HCNVR34 were associated with the reproduction traits in Bama Xiang pigs, including litter size and teat number. The phenotype of deletions of HCNVR10 and HCNVR34 were lower than normal in litter size and teat number. *NEURL1* and *SH3PXD2A* were located in HCNVR34. However, *NEURL1* and *SH3PXD2A*, possibly related to reproduction traits, have not been reported. The HCNVR34 (chr14 from 124353052 to 124685417) contained *NEURL1* and *SH3PXD2A* gene may be the candidate CNVR and candidate genes, which is related to litter size and teat number in Bama Xiang pigs.

### The Genes Related to Testis and Sperm

In GWAS, we found that nine genes were related to testis and sperm, including *CIB4, DRC1, HADHA, DMC1, DDX17, RAB23, HMGA1, SLC26A8*, and *ARRDC4*. In CNV detection, we found that *TUBB4B, STPG3, NRARP, LCN8, LCN12, LCN10*, and *CCDC183* were related to the testis and sperm. This result indicated that the sperm of Bama Xiang pigs were different than those of other pigs. Our previous study found that the sperm from Duroc improved the litter size of Bama Xiang pig sows compared to the sperm from Bama Xiang boars (59). We speculate that the reduced litter size in Bama Xiang is due to inbreeding; however, the quality of sperm should be studied further to ascertain the cause of the reduced litter size. Several genes related to testis and sperm were identified from litter-size traits, illustrating that the marks play an important role in Bama Xiang pigs. Bama Xiang boars' sperm quality could be enhanced to improve the litter size of Bama Xiang sows. However, inbreeding in parent and offspring should be deterred to improve the fertility of Bama Xiang pigs.

## Conclusions

In this study, we executed the GWAS, ROH analysis, and CNV detection using porcine 50K Beadchip in Bama Xiang pigs. A total of 29 candidate SNPs for seven litter-size traits and four teat-number traits were identified in Bama Xiang pigs using GWAS. Twelve candidate genes were identified in litter-size traits. A total of 9,322 ROHs were found, and the litter-size traits had a significant negative correlation with *F*_ROH_. A total of 3,317 CNVs were identified, of which 11 genes may be the candidates for reproduction traits. Sixteen genes related to the testis and sperm function were identified. Our results confirm that by using marker-assisted selection on 16 genes, we can improve the litter size of Bama Xiang boars.

## Data Availability Statement

The original contributions presented in the study are publicly available. This data can be found here: https://doi.org/10.6084/m9.figshare.19564270.v1.

## Ethics Statement

The animal study was reviewed and approved by Guangxi University.

## Author Contributions

JM and YL designed the study, conducted the experiments, and drafted original manuscripts. SZ, LF, WQ, and XC conducted parts of experiments. BX, BC, GL, and JL revised the manuscripts. All authors have read and agreed to the published version of the manuscript.

## Funding

This research was funded by the National Modern Agricultural Industrial Technology System (nycytxgxcxtd-15-01), the National Natural Science Foundation of China (81860150), and the Science and Technology Major Project of Guangxi (Guike-AA17292002).

## Conflict of Interest

The authors declare that the research was conducted in the absence of any commercial or financial relationships that could be construed as a potential conflict of interest.

## Publisher's Note

All claims expressed in this article are solely those of the authors and do not necessarily represent those of their affiliated organizations, or those of the publisher, the editors and the reviewers. Any product that may be evaluated in this article, or claim that may be made by its manufacturer, is not guaranteed or endorsed by the publisher.

## Supplementary Material

Supplementary Figure S1 The Manhattan plots obtained in the GWAS with teat number traits. The red line identifies the cut-off for suggestive significance. Red spots identify SNPs with suggestive significance. Traits, from the inner to outer lanes are the teat numbers on the left side (LTN), the teat numbers on the right side (RTN), total number of teats (TTN), and the minimum number of teats (MINTN).

Supplementary Figure S2 The top 10 QTLs in common CNVRs.
