# Supplementary material for: Genome-Wide Association Studies, Runs of Homozygosity Analysis, and Copy Number Variation Detection to Identify Reproduction-Related Genes in Bama Xiang Pigs
Source: Front Vet Sci. 2022 May 31;9:892815. doi: 10.3389/fvets.2022.892815 (PMC9195146; doi:10.3389/fvets.2022.892815)
Supplement: Supplementary file 1 [file Table_1.DOC]

Table S1 The traits of litter size and teat number in Bama Xiang pigs

| Traits | Number | Phenotype (Mean ± SD) | Traits | Number | Phenotype (Mean ± SD) |
| --- | --- | --- | --- | --- | --- |
| Average birth number (ABN) | 297 | 9.81±1.4 | birth numbers of parity 1th (BN1) | 297 | 8.19±1.9 |
| birth numbers of parity 2th (BN2) | 296 | 8.98±2.0 | birth numbers of parity 3th (BN3) | 292 | 9.65±2.1 |
| birth numbers of parity 4th (BN4) | 292 | 9.95±2.1 | birth numbers of parity 5th (BN5) | 290 | 10.22±1.9 |
| birth numbers of parity 6th (BN6) | 275 | 10.45±1.8 | birth numbers of parity 7th (BN7) | 223 | 10.42±1.7 |
| birth numbers of parity 8th (BN8) | 152 | 10.59±2.1 | birth numbers of parity 9th (BN9) | 82 | 10.62±2.0 |
| The maximum numbers of birth (MAXBN) | 297 | 11.98±1.8 | The minimum numbers of birth (MINBN) | 297 | 7.34±1.8 |
| The standard error of birth numbers (BNCV) | 297 | 0.16±0.1 | The teat numbers on the left side (LTN) | 296 | 5.83±0.7 |
| The teat numbers on the right side (RTN) | 296 | 5.86±0.8 | The minimum number of teats (MINTN) | 296 | 5.61±0.7 |
| Total number of teats (TTN) | 296 | 11.70±1.3 | The maximum number of teats (MAXTN) | 296 | 6.08±0.7 |

Table S2 The suggestive significance markers in the GWAS

| Traits | SSC | Marker position | Marker | Alleles | P-value |
| --- | --- | --- | --- | --- | --- |
| ABN | 4 | 16498305 | rs80960023 | G/A | 1.86E-05 |
| BN2 | 14 | 64213878 | rs342934270 | A/G | 1.96E-05 |
| BN2 | 3 | 119594382 | rs81376414 | A/G | 2.00E-05 |
| BN4 | 1 | 12024877 | rs80868278 | G/A | 3.29E-06 |
| BN5 | 7 | 88276755 | rs80955159 | A/C | 4.29E-06 |
| BN5 | 7 | 32915748 | rs80939400 | A/G | 5.68E-06 |
| BN5 | 7 | 88646105 | rs80785439 | G/A | 8.91E-06 |
| BN5 | 7 | 87714516 | rs80930654 | A/G | 1.00E-05 |
| BN5 | 7 | 88215937 | rs80880794 | G/A | 1.00E-05 |
| BN5 | 7 | 88363304 | rs80967544 | A/C | 1.38E-05 |
| BN5 | 7 | 88483539 | rs80994818 | A/G | 2.06E-05 |
| BN5 | 7 | 88557851 | rs80845197 | A/G | 2.06E-05 |
| BN5 | 7 | 48567905 | rs80815905 | A/G | 3.27E-05 |
| BN5 | 7 | 34978383 | rs342640079 | A/G | 3.82E-05 |
| BN6 | 17 | 64579005 | rs80959929 | A/C | 4.05E-06 |
| BN6 | 4 | 127546528 | rs80992798 | A/G | 1.57E-05 |
| BN6 | 4 | 124599055 | rs81306657 | G/A | 3.46E-05 |
| BN6 | 4 | 125219675 | rs325692606 | A/G | 3.46E-05 |
| BN9 | 16 | 18229086 | rs81253541 | A/C | 6.85E-06 |
| BN9 | 5 | 6865291 | rs341534304 | A/G | 1.54E-05 |
| MAXBN | 7 | 36755698 | rs327731712 | G/A | 2.42E-05 |
| MAXBN | 7 | 34997673 | rs345936691 | A/C | 2.45E-05 |
| MAXBN | 7 | 88363304 | rs80967544 | A/C | 3.05E-05 |
| MINTN | 14 | 30868951 | rs81450533 | G/A | 2.27E-05 |
| RTN | 1 | 58258682 | rs81354131 | C/A | 4.10E-05 |
| TTN | 14 | 30783101 | rs81235122 | G/A | 2.32E-05 |
| TTN | 14 | 30868951 | rs81450533 | G/A | 2.43E-05 |
| TTN | 1 | 7169568 | rs81290581 | C/A | 2.64E-05 |
| LTN | 14 | 30868951 | rs81450533 | G/A | 1.52E-05 |

Table S3 The genes were identified by Biomart program

| Trait | SNP | Gene |
| --- | --- | --- |
| ABN | 1 | *FAM91A1、ANXA13、FBXO32* |
| BN2 | 2 | *FAM89A、ARV1、TTC13、C1orf198、CAPN9、COG2、CIB4、C2orf70、EPT1、DRC1、ADGRF3、HADHB、HADHA、GAREM2* |
| BN4 | 1 | *ARID1B* |
| BN5 | 10 | *ADGRF5、ADGRF1、GRM4、HMGA1、ARRDC4、RAB23* |
| BN6 | 4 | *BMP7、COL11A1* |
| BN9 | 2 | *DMC1、DDX17、KDELR3、KCNJ4、TMEM184B、MAFF、PLA2G6、CDH6* |
| MAXBN | 1 | *GRM4、HMGA1、LHFPL5、SRPK1、SLC26A8、MAPK14、PNPLA1、C6orf222* |
| MINTN | 3 | *ZNF664、DNAH10、ATP6V0A2、TCTN2、EIF2B1、GTF2H3、DDX55* |
| RTN | 1 | *RIMS1* |
| TTN | 1 | *ZNF664、DNAH10、ATP6V0A2、TCTN2、EIF2B1、GTF2H3、DDX55* |
| LTN | 3 | *ZNF664、DNAH10、ATP6V0A2、TCTN2、EIF2B1、GTF2H3、DDX55* |

Table S4 The spearman coefficient between FROH and reproduction traits

| Team | ABN | BN1 | BN2 | BN3 | BN4 | BN5 | BN6 | BN7 | BN8 | BN9 |
| --- | --- | --- | --- | --- | --- | --- | --- | --- | --- | --- |
| F_ROH_ | -0.28** | -0.19** | -0.19** | -0.25** | -0.15* | -0.20** | -0.23** | -0.11 | -0.17* | -0.22** |
| Team | MAXBN | MINBN | BNSD | BNCV | LTN | RTN | TTN | MINTN | MAXTN |  |
| F_ROH_ | -0.22** | -0.25** | 0.03 | 0.14* | 0.02 | 0.04 | 0.04 | 0.02 | 0.04 |  |

Note: The ** means the highly significant relate between F_ROH_ and reproduction traits, the * means the significant relate between F_ROH_ and reproduction traits.

Table S5 The common CNVRs between cnvPartition and PennCNV

| CNVR | CHR | START | END | types |
| --- | --- | --- | --- | --- |
| CCNVR1 | 1 | 294714909 | 295500957 | mixed |
| CCNVR2 | 1 | 311585283 | 315307620 | mixed |
| CCNVR3 | 2 | 420244 | 1483817 | deletion |
| CCNVR4 | 2 | 13735970 | 15232176 | mixed |
| CCNVR5 | 2 | 62285470 | 62364174 | mixed |
| CCNVR6 | 2 | 62545133 | 63087289 | mixed |
| CCNVR7 | 2 | 158549163 | 162021324 | mixed |
| CCNVR8 | 3 | 142464557 | 144770913 | deletion |
| CCNVR9 | 5 | 20777007 | 22198667 | deletion |
| CCNVR10 | 5 | 103013980 | 103193204 | deletion |
| CCNVR11 | 7 | 23306243 | 23706793 | duplication |
| CCNVR12 | 7 | 25171813 | 27054480 | deletion |
| CCNVR13 | 7 | 81790769 | 82727444 | deletion |
| CCNVR14 | 9 | 56107708 | 56339476 | mixed |
| CCNVR15 | 10 | 5502952 | 6116151 | deletion |
| CCNVR16 | 11 | 38032989 | 41462103 | deletion |
| CCNVR17 | 12 | 29897886 | 30710965 | duplication |
| CCNVR18 | 13 | 120554530 | 123542574 | deletion |
| CCNVR19 | 13 | 163249576 | 165581857 | deletion |
| CCNVR20 | 13 | 170632687 | 172999223 | mixed |
| CCNVR21 | 15 | 25128485 | 25432887 | deletion |
| CCNVR22 | 15 | 108894219 | 109934030 | deletion |
| CCNVR23 | 18 | 15079181 | 16922697 | deletion |
| CCNVR24 | 18 | 27920480 | 29634538 | deletion |

Table S6 The higher frequency CNVRs

| chr | start | end | sample | type | Software | HCNVR Number |
| --- | --- | --- | --- | --- | --- | --- |
| 1 | 9324586 | 9599991 | 21 | deletion | cnvPartition | HCNVR1 |
| 1 | 248044630 | 248127147 | 23 | mixed | cnvPartition | HCNVR2 |
| 1 | 271202571 | 271509059 | 82 | deletion | cnvPartition | HCNVR3 |
| 1 | 308142318 | 308521887 | 25 | deletion | cnvPartition | HCNVR4 |
| 1 | 311585283 | 315307620 | 124 | mixed | cnvPartition | HCNVR5 |
| 2 | 432491 | 1483817 | 22 | mixed | cnvPartition | HCNVR6 |
| 2 | 9518463 | 9733580 | 46 | deletion | cnvPartition | HCNVR7 |
| 2 | 13735970 | 15232176 | 96 | mixed | cnvPartition | HCNVR8 |
| 2 | 67927979 | 68202594 | 43 | deletion | cnvPartition | HCNVR9 |
| 2 | 152455383 | 152748172 | 40 | deletion | cnvPartition | HCNVR10 |
| 2 | 158549163 | 162021324 | 188 | mixed | cnvPartition | HCNVR11 |
| 3 | 117770167 | 118132370 | 21 | deletion | cnvPartition | HCNVR12 |
| 4 | 10927526 | 11325432 | 53 | deletion | cnvPartition | HCNVR13 |
| 5 | 20777007 | 22198667 | 22 | mixed | cnvPartition | HCNVR14 |
| 5 | 62063128 | 62428176 | 58 | mixed | cnvPartition | HCNVR15 |
| 6 | 40183749 | 40660717 | 61 | mixed | cnvPartition | HCNVR16 |
| 6 | 86375688 | 87035352 | 29 | deletion | cnvPartition | HCNVR17 |
| 6 | 140414325 | 140483422 | 30 | deletion | cnvPartition | HCNVR18 |
| 7 | 25171813 | 27054480 | 64 | mixed | cnvPartition | HCNVR19 |
| 7 | 82179857 | 82727444 | 37 | mixed | cnvPartition | HCNVR20 |
| 7 | 111150278 | 111509134 | 26 | deletion | cnvPartition | HCNVR21 |
| 7 | 122877455 | 123228654 | 24 | deletion | cnvPartition | HCNVR22 |
| 9 | 56107708 | 56339476 | 38 | mixed | cnvPartition | HCNVR23 |
| 9 | 100713259 | 100971561 | 37 | deletion | cnvPartition | HCNVR24 |
| 10 | 22119210 | 23691720 | 23 | mixed | cnvPartition | HCNVR25 |
| 10 | 49018537 | 49481409 | 47 | deletion | cnvPartition | HCNVR26 |
| 11 | 15140002 | 16312635 | 43 | mixed | cnvPartition | HCNVR27 |
| 11 | 20780276 | 20909354 | 29 | mixed | cnvPartition | HCNVR28 |
| 11 | 70632869 | 71728833 | 50 | mixed | cnvPartition | HCNVR29 |
| 12 | 7397172 | 7738561 | 28 | deletion | cnvPartition | HCNVR30 |
| 13 | 20918664 | 21221095 | 86 | deletion | cnvPartition | HCNVR31 |
| 13 | 25799857 | 25919479 | 138 | deletion | cnvPartition | HCNVR32 |
| 13 | 170632687 | 172999223 | 27 | mixed | cnvPartition | HCNVR33 |
| 14 | 124353052 | 124685417 | 57 | deletion | cnvPartition | HCNVR34 |
| 15 | 31519100 | 31656437 | 23 | deletion | cnvPartition | HCNVR35 |
| 15 | 150772339 | 150964361 | 22 | deletion | cnvPartition | HCNVR36 |
| 16 | 1032442 | 1378859 | 70 | mixed | cnvPartition | HCNVR37 |
| 16 | 72041929 | 72312532 | 72 | deletion | cnvPartition | HCNVR38 |
| 18 | 1265384 | 1564813 | 25 | deletion | cnvPartition | HCNVR39 |
| 1 | 312198117 | 313171967 | 189 | mixed | PennCNV | HCNVR40 |
| 2 | 13735970 | 14603036 | 32 | mixed | PennCNV | HCNVR41 |
| 2 | 67927979 | 68202594 | 42 | mixed | PennCNV | HCNVR42 |
| 2 | 158619978 | 159432677 | 30 | mixed | PennCNV | HCNVR43 |
| 5 | 103046152 | 103157290 | 24 | deletion | PennCNV | HCNVR44 |
| 7 | 23306243 | 23706793 | 54 | mixed | PennCNV | HCNVR45 |
| 7 | 25400300 | 25975407 | 52 | mixed | PennCNV | HCNVR46 |
| 7 | 81790769 | 82629584 | 24 | mixed | PennCNV | HCNVR47 |
| 9 | 56107708 | 56339476 | 22 | mixed | PennCNV | HCNVR48 |
| 10 | 22659922 | 23181276 | 22 | deletion | PennCNV | HCNVR49 |
| 13 | 122198084 | 122415707 | 40 | deletion | PennCNV | HCNVR50 |

Table S7 The significance HCNVRs and suggestive significance HCNVRs in reproductive traits

| Trait | CNVR | P value | Trait | HCNVRR | P value |
| --- | --- | --- | --- | --- | --- |
| BNSD | CNV2 | 0.0005 | BNSD | CNV17 | 0.0040 |
| BNCV | CNV2 | 0.0044 | BNCV | CNV17 | 0.0107 |
| BN8 | CNV4 | 0.0054 | BN7 | CNV23 | 0.0073 |
| BN9 | CNV4 | 0.0176 | ABN | CNV34 | 0.0032 |
| BN8 | CNV6 | 0.0001 | MAXBN | CNV34 | 0.0032 |
| MINBN | CNV6 | 0.0015 | MAXTN | CNV34 | 0.0035 |
| BNCV | CNV6 | 0.0060 | TTN | CNV34 | 0.0038 |
| BN9 | CNV8 | 0.0005 | LTN | CNV34 | 0.0044 |
| BN5 | CNV10 | 0.0010 | MAXBN | CNV43 | 0.0103 |
| ABN | CNV10 | 0.0082 | BNCV | CNV47 | 0.0017 |
| BN4 | CNV10 | 0.0170 | BNSD | CNV47 | 0.0046 |
| MINBN | CNV10 | 0.0196 | BN3 | CNV49 | 0.0075 |
| BN6 | CNV13 | 0.0155 | MINBN | CNV16 | 0.0153 |

Table S8 The t-test of reproduction traits in HCNVR10 and HCNVR34

| Traits | CNVR12 (Mean±SD) | | | CNVR36 (Mean±SD) | | |
| --- | --- | --- | --- | --- | --- | --- |
|  | deletions | normal | P value | deletions | normal | P value |
| Number | 38 | 259 |  | 50 | 247 |  |
| ABN | 9.08±1.57 | 9.91±1.37 | 0.001 | 9.26±1.78 | 9.92±1.31 | 0.016 |
| BN1 | 8.21±2.01 | 8.18±1.92 | 0.931 | 7.96±1.82 | 8.23±1.96 | 0.368 |
| BN2 | 8.42±2.26 | 9.06±2.06 | 0.070 | 8.46±2.25 | 9.09±1.98 | 0.048 |
| BN3 | 9.16±2.25 | 9.73±2.01 | 0.110 | 9.17±2.32 | 9.75±1.98 | 0.072 |
| BN4 | 9.11±2.67 | 10.08±1.97 | 0.036 | 9.60±2.27 | 10.02±2.05 | 0.208 |
| BN5 | 9.13±2.04 | 10.38±1.82 | 0.000 | 9.98±2.00 | 10.26±1.88 | 0.348 |
| BN6 | 9.65±1.39 | 10.56±1.78 | 0.004 | 10.00±1.93 | 10.53±1.72 | 0.068 |
| BN7 | 9.62±1.74 | 10.54±1.66 | 0.06 | 9.88±1.81 | 10.52±1.66 | 0.044 |
| BN8 | 9.42±2.36 | 10.76±1.97 | 0.008 | 10.19±1.70 | 10.66±2.12 | 0.338 |
| BN9 | 10.50±1.88 | 10.64±2.08 | 0.824 | 10.88±1.81 | 10.59±2.07 | 0.714 |
| MAXBN | 11.26±2.00 | 12.08±1.72 | 0.008 | 11.28±2.02 | 12.12±1.69 | 0.002 |
| MINBN | 6.61±1.88 | 7.44±1.75 | 0.007 | 7.20±1.97 | 7.36±1.75 | 0.555 |
| BNSD | 1.53±0.49 | 1.53±0.55 | 0.983 | 1.41±0.42 | 1.56±0.56 | 0.037 |
| BNCV | 0.17±0.06 | 0.16±0.06 | 0.116 | 0.16±0.06 | 0.16±0.06 | 0.965 |
| LTN | 5.87±0.67 | 5.83±0.74 | 0.736 | 5.51±0.77 | 5.89±0.71 | 0.001 |
| RTN | 5.84±0.72 | 5.87±0.76 | 0.843 | 5.61±0.76 | 5.91±0.75 | 0.010 |
| MINTN | 5.69±0.67 | 5.60±0.74 | 0.525 | 5.37±0.76 | 5.66±0.72 | 0.010 |
| TTN | 11.71±1.25 | 11.69±1.29 | 0.940 | 11.12±1.33 | 11.81±1.24 | 0.001 |
| MAXTN | 6.03±0.68 | 6.09±0.68 | 0.571 | 5.78±0.72 | 6.15±0.65 | 0.000 |
