# Supplementary figures and images for: Genome-Wide Association Studies, Runs of Homozygosity Analysis, and Copy Number Variation Detection to Identify Reproduction-Related Genes in Bama Xiang Pigs
Source: Front Vet Sci. 2022 May 31;9:892815. doi: 10.3389/fvets.2022.892815 (PMC9195146; doi:10.3389/fvets.2022.892815)

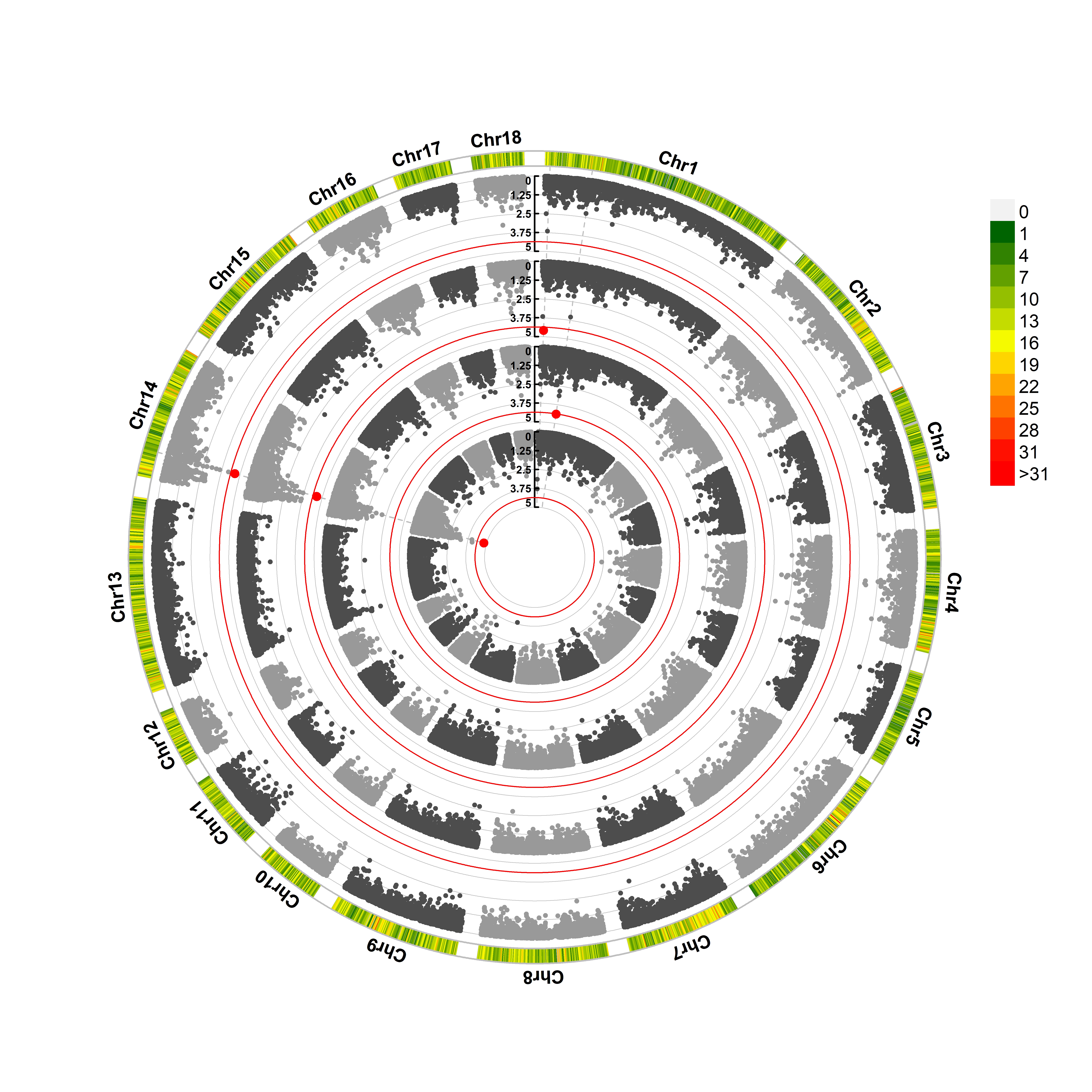

Supplement: Supplementary Figure S1 — The Manhattan plots obtained in the GWAS with teat number traits. The red line identifies the cut-off for suggestive significance. Red spots identify SNPs with suggestive significance. Traits, from the inner to outer lanes are the teat numbers on the left side (LTN), the teat numbers on the right side (RTN), total number of teats (TTN), and the minimum number of teats (MINTN). [file Image_1.JPEG]

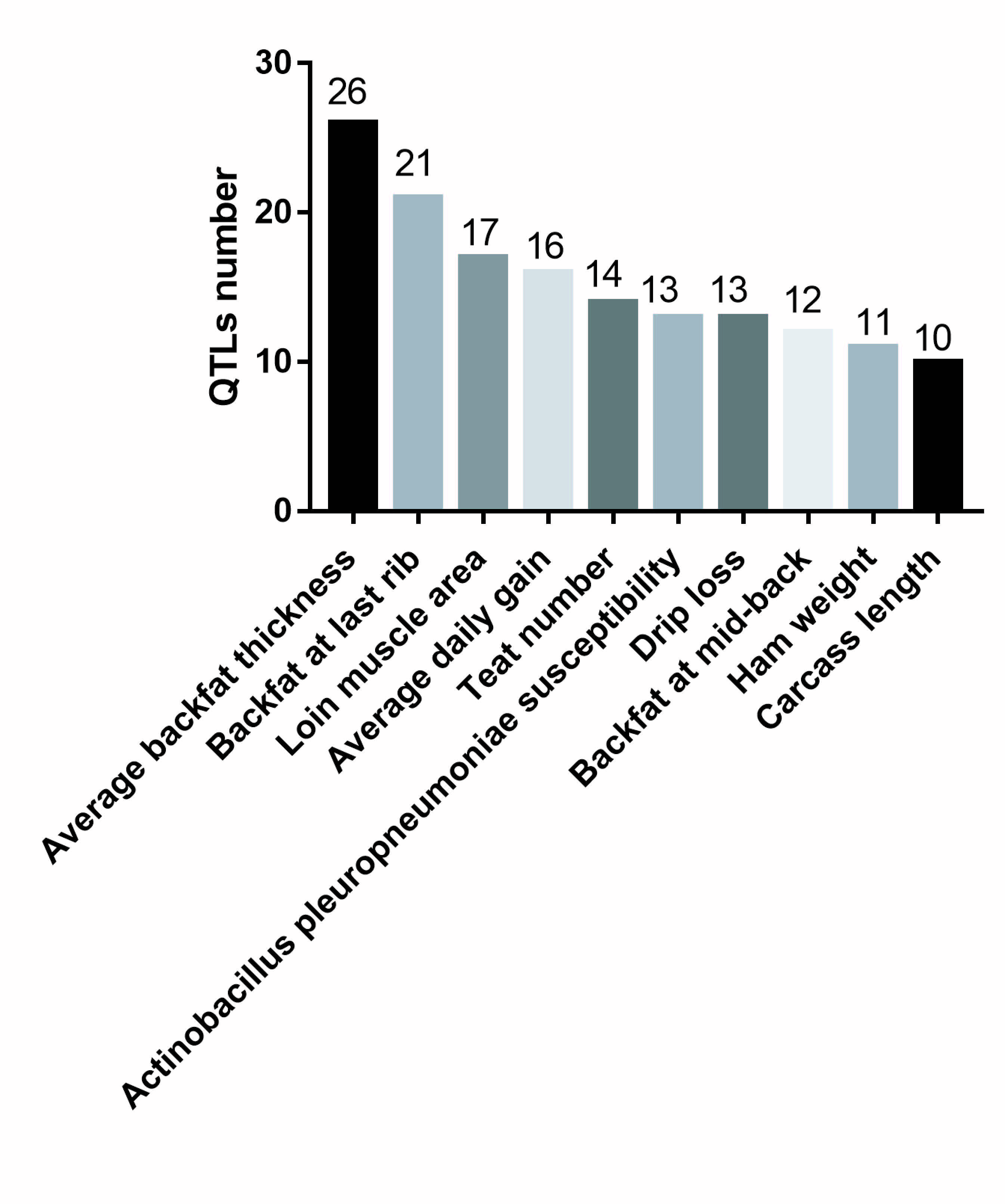

Supplement: Supplementary Figure S2 — The top 10 QTLs in common CNVRs. [file Image_2.JPEG]
